# Early prediction of contrast-induced acute kidney injury by a "bedside" assessment of Neutrophil Gelatinase-Associated Lipocalin during elective percutaneous coronary interventions

**DOI:** 10.1371/journal.pone.0197833

**Published:** 2018-05-23

**Authors:** Annunziata Nusca, Marco Miglionico, Claudio Proscia, Laura Ragni, Massimiliano Carassiti, Francesca Lassandro Pepe, Germano Di Sciascio

**Affiliations:** 1 Unit of Cardiac Sciences, Campus Bio-Medico University of Rome, Rome, Italy; 2 Unit of Anesthesiology, Critical Care and Pain Medicine, Campus Bio-Medico University of Rome, Rome, Italy; National Institute of Health, ITALY

## Abstract

Contrast-induced acute kidney injury (CI-AKI) is a serious complication during percutaneous coronary interventions (PCI). Currently, the diagnosis of CI-AKI relies on serum creatinine (SCr) that is however affected by several limitations potentially leading to delayed or missed diagnoses. In this study we examined the diagnostic accuracy of a “bedside” measurement of plasma Neutrophil Gelatinase-Associated Lipocalin (NGAL) in the early detection of CI-AKI in 97 patients undergoing elective PCI. The overall incidence of CI-AKI was 3%. A significant positive correlation was observed between 6-hours NGAL and post-PCI SCr (r = 0.339, p = 0.004) and a significant negative correlation between 6-hours NGAL and post-PCI CrCl (r = -0.303, p = 0.010). In patients with post-PCI SCr increase > 0.24 mg/dl (median SCr absolute increase), delta NGAL 0–6 hours and 6-hours NGAL values were higher compared with patients with SCr elevation below the defined threshold (p = 0.049 and p = 0.056). The ROC analysis showed that a 6 hours NGAL value > 96 ng/ml significantly predicted an absolute SCr increase > 0.24 mg/dl after contrast exposure with sensitivity of 53% and specificity of 74% (AUC 0.819, 95% CI: 0.656 to 0.983, p = 0.005). The use of bedside NGAL assessment may significantly hasten diagnosis and treatment of CI-AKI, with remarkable clinical prognostic consequences.

## Introduction

Percutaneous coronary intervention (PCI) is part of standard therapy in patients presenting with stable and unstable coronary syndromes. However, with growing technical procedural improvements, also the risk profile of patients undergoing a PCI procedure has increased considerably over time, as the complexity of lesions being treated [1]. This change in patient characteristics in terms of age, cardiovascular risk factors and co-morbidities, may significantly influence long-term outcome as well as the incidence of peri-procedural complications as contrast-induced acute kidney injury (CI-AKI) and myocardial damage [2–4]. CI-AKI is the third leading cause of hospital-acquired acute kidney injury and its incidence ranges from 1–2% in patients without co-morbidities up to 25% in high-risk populations (patients with chronic kidney disease, diabetes, congestive heart failure); moreover, it has been consistently associated with prolonged length of stay, persistent kidney damage, rarely requiring dialysis, and all-cause mortality [5,6]. Therefore, there is a clear need, in clinical practice, to early identify those patients at high risk for post-procedure renal function deterioration, in order to guide prompt therapeutic decisions and improve outcomes. Currently, the diagnosis of CI-AKI relies on serum creatinine (SCr) and urinary output [7], although it is widely acknowledged that creatinine has several limitations, including its delayed response to kidney injury [8]. In the last decade, many biomarkers have been under investigation for their sensitivity and specificity in identifying early kidney cellular damage, rather than only functional deterioration, among which Neutrophil Gelatinase-Associated Lipocalin (NGAL), Kidney Injury Molecule-1 (KIM-1) and interleukin-18 [9].

NGAL is one of the earliest and most prominently induced genes in the kidney after ischemic or nephrotoxic injury in animal models [10]. Moreover, it is released and easily detected in the blood and urine of patients in the early stages after acute kidney injury due to different clinical conditions [11–15]. Emerging evidence shows that patients with elevated NGAL, in the absence of creatinine-based criteria for AKI, carry an increased risk of adverse events including need for renal replacement therapy and death [16,17]. Finally, NGAL has been demonstrated overexpressed during myocardial ischemia [18–20]; interestingly, high plasmatic NGAL levels independently predict all-cause mortality and major adverse cardiac events in ST-segment elevation myocardial infarction patients treated with primary PCI [21].

Although the diagnostic and prognostic importance of NGAL in CI-AKI is emerging in literature, little is known about the use of “point-of-care” platform for measurement of plasmatic levels of this biomarker in clinical practice, offering rapid and as accurate results as standardized laboratory testing in a remarkable shorter time interval; moreover, the only few studies with this laboratory tool have been conducted in the emergency department setting [22, 23]. Thus, the aim of our study is to evaluate the diagnostic accuracy of "bedside" NGAL assessment, compared with the standard SCr dosage, in the early detection of CI-AKI and, compared with standard cardiac troponin measurement, in the early identification of patients suffering from peri-procedural myocardial damage after coronary stenting in the non-acute setting.

## Methods

### Study population

We prospectively enrolled 97 consecutive patients undergoing PCI at our Institution between September 2016 to June 2017. Exclusion criteria were: chronic renal failure (glomerular filtration rate–GFR < 60 ml/min/1.73m2); left ventricular ejection fraction <30%; primary intervention for acute myocardial infarction; non-ST segment elevation acute coronary syndromes within the past 48 hours; coexistent immunological, inflammatory or neoplastic disease at the time of enrolment; severe pulmonary disease; thrombocytopenia (platelets < 70 × 103/ml). Coronary intervention was performed with standard technique. Aspirin 100 mg and clopidogrel 75 mg were administered in all patients before the procedure; moreover, in those not already on antiplatelet agents (56%, 54 patients) a loading dose of antiplatelet agents (aspirin 100–325 mg and clopidogrel 600 mg) was given at least two hours before PCI. During the procedure, all patients received a bolus of unfractionated heparin 70–100 IU/Kg body weight in order to obtain an activated clotting time > 300 seconds (or between 200 and 300 seconds, if GPIIb/IIIa were administered for high-thrombotic risk angioplasty). All enrolled patients underwent intravenous peri-procedural hydration with normal saline (1 ml/hour/kg body weight for at least 12 hours before and 24 hours after intervention). No specific protocol for periprocedural use of other potentially renal-protective agents was used. All interventions were performed with a nonionic, low-osmolar (915 mOsm/kg), iodinated contrast agent (iobitridol, Xenetix, Guerbet, Roissy CdG Cedex, France). The procedure was considered successful if there was <30% residual stenosis in the target lesion, with TIMI (Thrombolysis in Myocardial Infarction) grade III flow and in the absence of major in-hospital complications: death, myocardial infarction or urgent coronary revascularization (re-PCI or coronary artery bypass graft). All subjects enrolled in this study provided written informed consent. The Campus Bio-Medico University Ethical Committee approved the study.

### Laboratory assays

SCr was measured at hospital admission, 6 and 24 hours after PCI and thereafter if clinically indicated; for CI-AKI detection, the postprocedure peak value was used. The creatinine clearance (CrCl) was estimated by means of glomerular filtration rate (GFR), calculated according to the Cockcroft and Gault formula: CrCl ([140 age] weight/ serum creatinine 72) with adjustment for female gender (CrClfemale CrCl 0.85) [24]. CI-AKI was defined as an absolute increase in SCr ≥ 0.3 mg/dl compared to baseline within 24–48 hours after contrast administration [7].

Whole blood samples were also collected before and 6 hours after the procedure for the determination of NGAL by using the bedside NGAL kit (Alere Triage® CardioRenal Panel–San Diego, California), a point-of-care fluorescence immunoassay, whose on-board algorithm is capable of reporting a test result within 15 to 25 minutes. Creatine kinase–MB (CK-MB) and troponin I (TnI) levels were measured at the time of intervention, 6 and 24 hours after PCI, and thereafter if clinically indicated, according to standard enzymatic procedures (LOCI™ immunochemiluminometric assay—SIEMENS). The laboratory upper limits of normal (ULN, the 99th percentile of normal population with a total imprecision of 10%) were 3.6 ng/ml for CK-MB and 0.05 ng/ml for TnI. Peri-procedural myocardial damage (PMI) was defined by elevation of TnI (> 5 x 99th percentile Upper Reference Limit -URL) in patients with normal baseline values (≤ 99th percentile URL) or an increase of TnI > 20% if the baseline values were elevated, in addition to either symptoms suggestive of myocardial ischaemia or new ischaemic ECG changes or angiographic findings consistent with a procedural complication or imaging demonstration of new loss of viable myocardium or new regional wall motion abnormality, according to the accepted third universal definition of myocardial infarction [25].

### Statistical analysis

Data are presented as frequencies and percentages for categorical variables and mean ± SD or median and first and third quartiles, when appropriate, for continuous variables. The Kolmogorov-Smirnov test was used to identify potential deviations from the normal distribution. Correlation between normally distributed continuous variables was determined by Pearson correlation coefficients, whereas Spearman correlation coefficients were used to analyze not normally distributed variables. Continuous variables were compared by *t* test for normally distributed values; otherwise the Mann-Whitney *U* test was used. Ability of the assay to discriminate between patients with and without post-PCI SCr increase was evaluated by receiver-operating characteristic (ROC) curve analysis. The optimal cut-off value was calculated by determining the NGAL value providing the greatest sum of sensitivity and specificity. Odds ratio (OR) and 95% confidence intervals (CIs) investigating the independent predictive role of NGAL increase on the occurrence of the SCr elevation was assessed by logistic regression analysis. A value of p<0.05 was considered statistically significant. Statistical analysis was performed using the SPSS 16.0 package for Windows.

## Results

Clinical, demographic and laboratory baseline characteristics of the study population are shown in Table 1 and S1 Dataset. Median age of the study population was 67 ± 9 years. Diabetes mellitus was present in 37 patients (38%) of which 14 patients were on insulin therapy. The majority of patients (77%) underwent coronary angiography for stable angina, whereas 22 patients were studied for a non-ST segment elevation acute coronary syndrome occurred > 48 hours before admission. History of previous myocardial infarction was reported in 23 patients (24%), while 38% of the entire population had previously undergone percutaneous myocardial revascularization. Median left ventricle ejection fraction before the procedure was 56 ± 10% assessed by transthoracic echocardiography or left ventriculography. Median pre-procedural NGAL levels were 99.6 mg/dl ± 53.1 mg/dl and pre-procedural blood SCr levels were 0.94 mg/dl ± 0.20 mg/dl with a median GFR of 87.8 ± 31.7 ml/min. Procedural characteristics are reported in Table 2. Multivessel disease was present in 61% of the entire population with a relatively high presence of B2/C lesions (44%). Procedural success was obtained in 96% of all cases, with median amount of administered contrast medium of 157.9 ml ± 65.9 ml.

**Table 1:** Clinical, demographic and laboratory baseline characteristics of the study population.

Variable (unit)	N = 97
Age (years)	67 ± 9
Male gender	77 (79)
BMI (Kg/cm^2^)	27.7 ± 4.0
Hypertension	80 (82)
Dyslipidemia	65 (67)
Diabetes Mellitus type 2	37 (38)
Insulin treated	14 (14)
Smokers	61 (63)
Clinical presentation	
Stable angina	75 (77)
Unstable angina/NSTEMI	22 (23)
Previous MI	23 (24)
Previous PCI	38 (39)
Previous CABG	4 (4)
LVEF (%)	56 ± 10
FGL (mg/dl)	101.8 ± 23.2
Creatinine (mg/dl)	0.94 ± 0.20
CrCl (ml/min)	87.8 ± 31.7
NGAL (ng/ml)	99.6 ± 53.1
CK-MB (ng/ml)	0.8 ± 0.2
Troponin I (ng/ml)	0.5 ± 4.1

Values are indicated as mean ± standard deviation (SD) or n (%). BMI, body mass index; CK-MB, creatine-kinase muscle-brain isoform; CrCl, creatinine clearance; FGL, fasting blood glucose levels; NGAL, neutrophil gelatinase associated lipocalin; NSTEMI, non-ST segment elevation myocardial infarction.

**Table 2:** Procedural characteristics.

Characteristics (unit)	
Multivessel disease	59 (61)
LM	1 (1)
LAD	48 (50)
LCX	26 (27)
RCA	21 (21)
SVG	1 (1)
Complete revascularization	49 (51)
% of stenosis	78 ± 8
Type of lesion	
A/B1	54 (56)
B2/C	43 (44)
N. stents/patient	1.1 ± 1.0
N. DES	38 (39)
Stent diameter (mm)	3.1 ± 0.4
Stent length (mm)	19.0 ± 5.5
Direct stenting	41 (42)
Procedural success	93 (96)
Contrast medium (ml)	157.9 ± 65.9

Values are indicated as mean ± standard deviation (SD) or n (%). DES, drug-eluting stent; LAD, left anterior descending; LCX, left circumflex; LM, left main; RCA, right coronary artery; SVG, saphenous venous graft.

**Fig 1:**
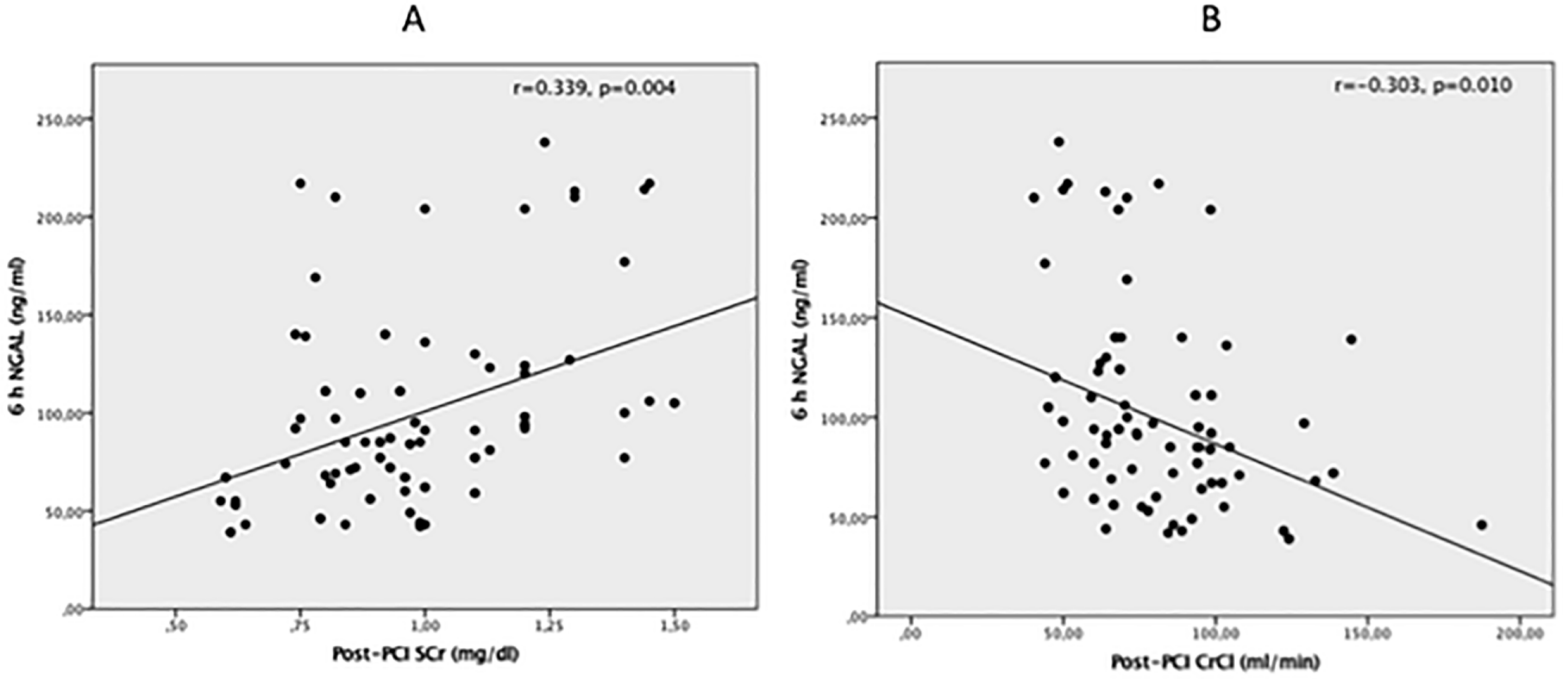
Correlation between 6-hours post-PCI NGAL and post-PCI SCr (r = 0.339, p = 0.004) (A) and 6-hours post-PCI NGAL and post-PCI CrCl (r = -0.303, p = 0.010) (B).

**Fig 2:**
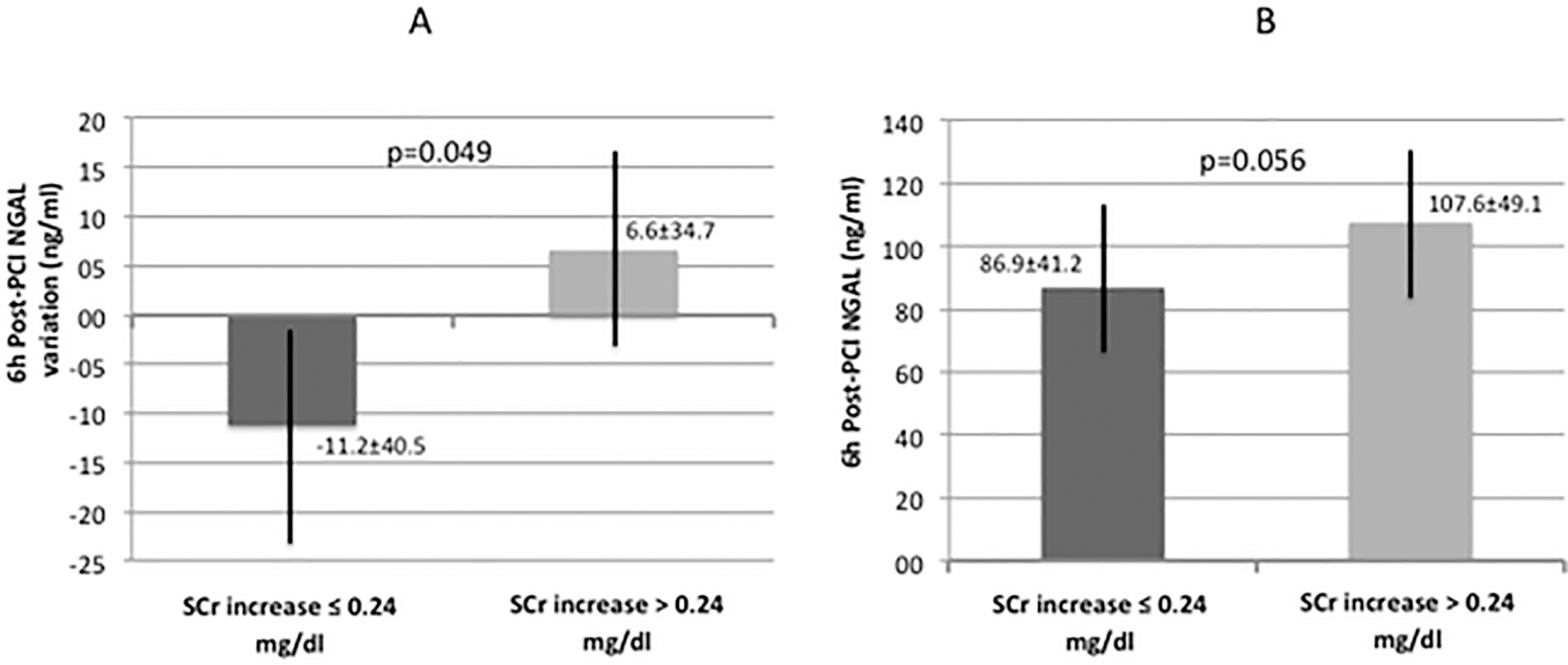
6h post-PCI NGAL variation (A) and 6h post-PCI NGAL absolute values (b) in patients with and without SCr increase > 0.24 mg/dl.

**Fig 3:**
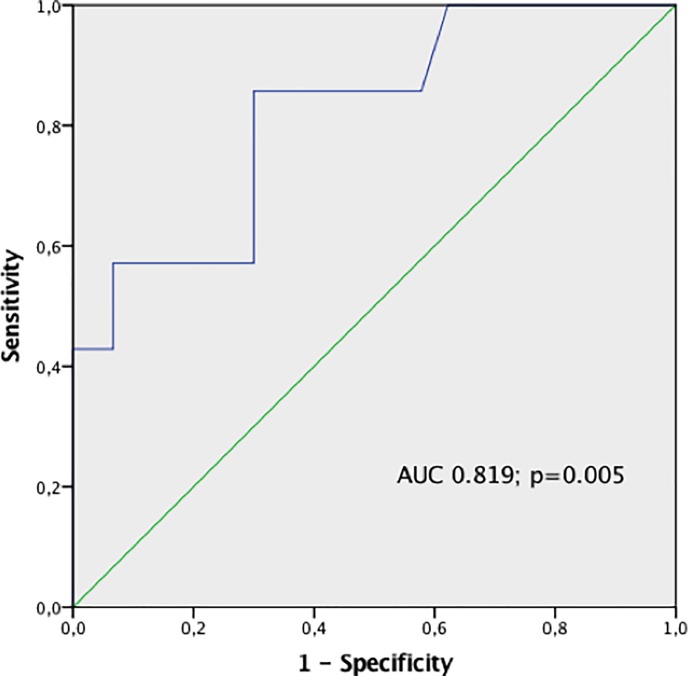
ROC curve analysis. An increase in 6h post-PCI NGAL > 96 ng/ml significantly predicts an absolute SCr increase > 0.24 mg/dl after contrast exposure with sensitivity of 53% and specificity of 74% (AUC 0.819, 95% CI: 0.656 to 0.983, p = 0.005) and with and OR of 3.15 (p = 0.023).

The overall incidence of CI-AKI in the study population was 3%. A significant increase in post-PCI SCr (0.94 mg/dl ± 0.20 mg/dl vs 0.97 mg/dl ± 0.20 mg/dl; p = 0.009) and a significant decrease in post-PCI CrCl (87.8 ml/min ± 31.7 ml/min vs 85.0 ml/min ± 28.3 ml/min; p = 0.014) were observed compared with baseline. We found a significant positive correlation between 6-hours post-PCI NGAL and post-PCI SCr (r = 0.339, p = 0.004) and a significant negative correlation between 6-hours NGAL and post-PCI CrCl (r = -0.303, p = 0.010) (Fig 1A and Fig 1B). We estimated the median post-PCI SCr absolute increase (ΔSCr) as 0.24 mg/dl and divided the study population in two groups: patients with ΔSCr > 0.24 mg/dl and those with ΔSCr < 0.24 mg/dl. In the first group (ΔSCr > 0.24 mg/dl) NGAL increase was significantly higher compared with patients with SCr elevation below the previously defined threshold (6.6 ± 34.7 vs -11.2 ± 40.5 ng/ml, p = 0.049) (Fig 2A). Similar results were observed for the 6 hours post-PCI absolute NGAL levels: in patients with ΔSCr > 0.24 mg/dl, NGAL concentrations were 107.6 ± 49.1 versus 86.9 ± 41.2 ng/ml in the other group (p = 0.056) (Fig 2B). The ROC analysis showed that a 6 hours NGAL value > 96 ng/ml significantly predicted an absolute SCr increase > 0.24 mg/dl after contrast exposure with sensitivity of 53% and specificity of 74% (AUC 0.819, 95% CI: 0.656 to 0.983, p = 0.005) (Fig 3); logistic regression analysis confirmed the predictive value of post-PCI NGAL values (OR = 3.15, p = 0.023).

In a similar fashion, a significant correlation between 6-hours post-PCI NGAL values and 24-hours troponin I levels was observed (r = 0.327, p = 0.005). Eighteen patients (19%) developed PMI, showing both higher 6-hours post-PCI NGAL values (111.0 ± 39.0 ng/ml versus 93.3 ± 47.1 ng/ml; p = 0.158) and higher absolute NGAL increase (ΔNGAL, +15.6 ± 15.7 ng/ml versus -7.3 ± 41.2 ng/ml; p = 0.001) compared with patients who did not develop this complication.

## Discussion

To our knowledge, this is one of the first studies in the literature assessing the role of a “point-of-care” assay for measurement of serum NGAL as an early marker of CI-AKI after elective PCI, compared with established markers of acute nephropathy. The main findings of this study denote that: (a) the incidence of CI-AKI was comparable to that reported in the literature for low-risk patients undergoing elective coronary procedures (around 3.5%) [5], with a significant increase in the levels of SCr and a consequent significant decrease in CrCl 24–48 hours after contrast media administration in the overall study population compared with baseline; (b) 6 hours post-PCI NGAL values showed a significant positive correlation with post-PCI SCr increase and CrCl decrease; (c) both ΔNGAL and absolute post-PCI values resulted to be higher in patients whose creatinine rise was above the overall population median SCr increase (ΔSCr > 0.24 mg/dl); (d) a 6 hours post-PCI NGAL of 96 ng/ml was found to be a cut-off value to predict an absolute SCr increase > 0.24 mg/dl after contrast exposure; (e) a significant correlation was observed between 6-hours NGAL values and 24-hours troponin I levels, with higher post-PCI NGAL values and absolute NGAL increase in patients who developed PMI.

The reported incidence of CI-AKI varies widely across the literature depending on the definition, patient population, baseline risk factors and clinical setting. The incidence of CI-AKI has decreased over the past decade from a general incidence of around 15% to 7%, owing to a better awareness of the problem, better risk prevention measures and improved low-osmolar iodinated contrast media, with less renal toxicity [3]. Certain comorbidities (diabetes mellitus, chronic renal failure with proteinuria, hypertension, and dehydration), acute clinical setting and nephrotoxic co-medications further increase the risk of CI-AKI following angiographic procedures [26]. CI-AKI has been observed in < 3.5% of low-risk patients undergoing elective PCI, with a dramatic increase up to 15–19% in patients with acute coronary syndromes [27, 28]. In our study, we defined CI-AKI using the first definition of the Acute Kidney Injury Network (AKIN) criteria: an abrupt reduction in kidney function defined as an absolute increase in serum creatinine of more than or equal to 0.3 mg/dl within 48 hours [7]. According to this definition and others currently used in the literature, serial measurement of SCr is, to date, the only widely accepted method for detection of functional renal impairment after contrast media administration. However it is undermined by several limitations: SCr is dependent on age, gender, body mass index, basal GFR and although it starts rising within the first 24 hours from contrast exposure, it peaks at 2–5 days, returning to baseline in 2–3 weeks; moreover, most importantly, when SCr reaches its post-procedural peak, a notable impairment (i.e. > 50%) of renal glomerular function has already developed [29]. In addition, creatinine excreted in the urine is not solely a result of glomerular filtration but also a result of renal tubular secretion; this means that changes in SCr will underestimate the true fall in GFR. All these limitations may translate into a delay of treatment of patients who will ultimately develop CI-AKI or, even worse, to missed diagnoses of this complication in patients discharged before the peak of SCr is reached. Notably, it could also lead to a prolonged in-hospital stay with considerable logistical and financial consequences. Hence, the need for early and more sensitive biomarkers, such as NGAL, possibly identifying kidney tubular damage before functional impairment develops, is evident. The use of such biomarkers may allow to the identification of a new category of patients with “subclinical AKI” (i.e., an increase in damage markers alone without simultaneous loss of kidney function), with important therapeutic and prognostic implications [30]. NGAL assessment may be even more useful in a population same as enrolled in our study, a low-intermediate risk cohort undergoing elective PCI and discharged within 24–48 hours after the procedure, with low or possibly underestimated incidence of functional kidney impairment. Furthermore, it has been reported that NGAL increase is an independent predictor of unfavorable outcome and worse prognosis, irrespective of the presence of functional damage. Haase et al [16] conducted a multicenter analysis of pooled data to explore the prognostic value of acute kidney injury detected by urine and plasma NGAL. They found that a positive NGAL finding carried a similar risk of adverse outcome than a positive creatinine finding. Moreover, they noticed that NGAL(+)/SCr(-) tests identified approximately 40% more acute kidney injury cases than SCr(+) alone and that these patients were at greater risk of longer intensive care unit and hospital stay, renal replacement therapy and death compared with control subjects. Furthermore, Nickolas et al evaluated the diagnostic and prognostic value of urinary biomarkers of intrinsic acute kidney injury (among which NGAL) in patients admitted to the emergency department [17]. They found that urinary NGAL was elevated in intrinsic AKI and predictive of the severity and duration of renal failure. Moreover, urinary NGAL predicted a composite outcome of dialysis initiation and death during hospitalization.

In our study, the post-procedural delta NGAL significantly correlated with post-PCI SCr increase and CrCl reduction and, more interestingly, both the post-PCI NGAL increase and the 6-hours absolute values were significantly higher in patients who developed a SCr elevation greater than 0.24 mg/dl (i.e. 0.06 mg/dl below the threshold for identification of CI-AKI according to the AKIN criteria). In a similar fashion, Padhy et al [31] previously observed that in a nested case control study, serum NGAL by ELISA increased sharply at 4 hours after coronary angioplasty to gradually decline to near normal level at 48 h in AKI cases (intended as a rise in SCr of at least 0.5 mg/dl from the baseline value at 48 h after PCI), while it did not increase significantly in non-AKI patients [30]. In their prospective study, Tasanarong and colleagues [6] found that urine NGAL assessed by ELISA at 6 hours and delta NGAL 0–6 hours post coronary procedures were good biomarkers for early diagnosis of CI-AKI and had some value in grading its severity; a significant difference in NGAL increase was observed among different stages of CI-AKI severity, with higher concentrations in more severe kidney injury. Finally, Quintavalle et al [32] assessed the role of urine NGAL and serum NGAL at 2, 6, 24, and 48 hours after contrast media exposure for CI-AKI detection (defined as SCr increase ≥0.3 mg/dl) in 458 high-risk patients (GFR ≤ 30 ml/min) undergoing coronary or peripheral angiography or angioplasty. They found that optimal thresholds for CI-AKI occurred at 6 hours for both urine NGAL (≥20 ng/ml; 97% negative predictive value and 27% positive predictive value) and serum NGAL (≥179 ng/ml; 93% negative predictive value and 20% positive predictive value). No patients with NGAL below these thresholds developed CI-AKI. Moreover, serum NGAL ≥179 ng/mL at 6 hours was an independent predictor of 1-year major adverse events (death, dialysis, nonfatal myocardial infarction, sustained kidney injury, and myocardial revascularization). Our cut-off value of serum NGAL as SCr increase predictor is markedly lower than that found by Quintavalle and colleagues; however, it must be noted that their larger study population (compared with our cohort) was at high-risk for CI-AKI development and had different baseline characteristics, in that it had an estimated GFR ≤ 30 mL/min, which would have been an exclusion criterion for our study. Despite the increased interest in the role of NGAL in the early detection of renal damage after contrast exposure, few studies evaluated the additional benefit of a “point-of-care” assay to measure plasma levels of these biomarker, thus allowing a quick (about 20 minutes) evaluation of the patient’s risk of developing CI-AKI. Soto et al have used bedside NGAL as a marker of AKI in patients admitted to the emergency department [22]; plasma NGAL discriminated AKI from normal function and transient azotemia. Similarly, Shapiro and colleagues found that plasma NGAL concentrations measured on presentation to the emergency department in patients with suspected sepsis were associated with the development of acute kidney injury [23].

Finally, we observed a significant correlation between 6-hours post-PCI NGAL values and 24-hours troponin I levels, with significantly higher 6-hours NGAL values and NGAL increase in patients who developed PMI. Several studies in the literature have described the overexpression of NGAL in myocardial ischemia animal models [18,19], as well as in patients with stable coronary artery disease and post-myocardial infarction heart failure [20]. Lindberg et al found that high plasma NGAL independently predicts all-cause mortality and major adverse events in patients receiving primary PCI for ST-segment elevation myocardial infarction [21]. NGAL has been documented in the vascular media, both in its free form and in a complex with metalloproteinase-9; moreover, the formation of this complex NGAL/MMP-9 prevents degradation of MMP-9 and reinforces its proteolytic activity, promoting the destabilization of atherosclerotic plaques [33]. Also, NGAL may play a role in the remodeling of the left ventricle in heart failure and specifically it may be involved with metalloproteinases in the changes occurring in the extracellular matrix [34].

### Study limitations

Some limitations of the present study have to be acknowledged. The relatively small sample size may undoubtedly limit the value of our findings. The incidence of CI-AKI may be probably underestimated since in most patients we obtained SCr levels 24 hours after contrast exposure. However, the relatively low incidence of CI-AKI in our study population may be also explained by the low-intermediate risk (elective procedural setting, stable syndromes in the majority of cases, exclusion of patients with chronic renal failure and heart failure). Moreover, all patients were hydrated with normal saline 1 ml/hour/kg body weight for at least 12 hours before and 24 hours after intervention (that is, up to date, the only preventive measure recommended, along with the use of low-osmolar or iso-osmolar contrast media). In our study we evaluated plasma NGAL assessed by a bedside assay, without any evaluation of urine NGAL. Urine NGAL is mainly produced by the distal nephron after injury and is directly secreted into the urine. In contrast, although serum NGAL probably arises predominantly from injured thick ascending tubules and collecting ducts, it is a product of multiple sources and might be a good biomarker of inflammation. In this view, serum NGAL values may be affected by several coexisting variables, such as chronic kidney disease, systemic infections, inflammatory conditions, anemia, and hypoxia. Hence, our study exclusion criteria comprehended coexistent immunological, inflammatory or neoplastic disease at the time of enrolment. Moreover, a recent systematic review and meta-analysis supported that the diagnostic accuracy of plasma/serum NGAL was similar to that of urine NGAL [16].

## Conclusion

To our knowledge, this is the first study to assess the role of a “point-of-care” assay designed for bedside plasma NGAL measurement in the early detection of contrast-induced acute kidney injury after elective percutaneous coronary intervention. The use of this tool may obviously significantly improve diagnosis and treatment of patients who develop this unfavourable complication, leading to remarkable clinical prognostic consequences.

## Supporting information

S1 Dataset Excel database with demographic and clinical features of the enrolled population. (XLSX)
